# Sulphur limitation provokes physiological and leaf proteome changes in oilseed rape that lead to perturbation of sulphur, carbon and oxidative metabolisms

**DOI:** 10.1186/1471-2229-13-23

**Published:** 2013-02-07

**Authors:** Philippe D’Hooghe, Sacha Escamez, Jacques Trouverie, Jean-Christophe Avice

**Affiliations:** 1 UMR INRA-UCBN 950 Écophysiologie Végétale, Agronomie & nutritions NCS, Institut de Biologie Fondamentale et Appliquée, Université de Caen Basse-Normandie, Esplanade de la Paix, CS 14032, Caen Cedex F-14032, France

**Keywords:** Sulphur limitation, Oilseed rape, Leaf proteome, Carbon metabolism, Oxidative stress

## Abstract

**Background:**

The decline in industrial emissions of sulphur (S) has led to a sulphate depletion in soil resulting in an alteration of crop performance. In oilseed rape, an S deficiency dramatically reduced the seed yield and/or quality. Paradoxically, little is known about the impact of sulphate limitation on oilseed rape leaf metabolism, despite it being a key determinant of growth. In order to identify the metabolic processes involved in the oilseed rape response to S restriction, an analysis of the young leaf proteome combined with a physiological study was carried out at the vegetative stage.

**Results:**

S limitation does not significantly reduce the total shoot biomass but inhibits growth and photosynthesis of young leaves. This photosynthesis decline is not due to a decrease in chlorophyll content, which remains similar to Control. The increase in anthocyanins and H_2_O_2_ content in young leaves of S-limited plants suggests that S restriction leads to an oxidative stress. Proteomic analysis at 35 d of S limitation also revealed the induction of 12-oxophitodienoate reductase and ACC synthase, respectively involved in jasmonate and ethylene biosynthesis, two phytohormones that could be implicated in oxidative stress. Proteins involved in photosynthesis and carbon metabolism were also modulated by S restriction. In particular, the decrease in plastocyanin and ferredoxin–NADP reductase suggests that H_2_O_2_ accumulation is associated with perturbation of the photosynthetic electron transport chain. The accumulation of chloroplastic Cu-Zn SOD reinforces the idea that an oxidative stress probably occurs in the chloroplast. Proteomic results suggest that the maintenance of chlorophyll in S-limited conditions is related to an accumulation of Water Soluble Chlorophyll binding Proteins, involved in the protection of chlorophyll against ROS. The accumulation of the catalytic α–subunit of chloroplastic ATP synthase suggests that energy production is maintained.

**Conclusion:**

S limitation leads to photosynthesis and carbon metabolism disturbances that could be responsible for the oxidative stress observed in the young leaves of oilseed rape. Despite this, induction of proteins involved in oxidative stress resistance and energy production shows that the leaf capacity to capture and use photosynthetic active radiations for ATP production remains efficient for as long as possible.

## Background

Crop plants take up sulphur (S) mainly in the form of sulphate and assimilate it into many compounds such as cysteine, methionine, glutathione (GSH), co–enzymes and vitamins. In addition, S is present within many plant secondary metabolites possessing various functions in plant metabolism [1]. Compared with other crops such as cereals or legumes, oilseed rape (*Brassica napus* L.) is particularly sensitive to S limitation because it has a high demand for S [2]. The decline in industrial emissions of SO_2_ leads to a depletion of sulphate (SO_4_^2–^) in soil, which impacts on oilseed rape growth and on both grain yield and oil quality [3]. Recent transcriptomic and metabolomic approaches have shown that alterations in the expression levels of numerous genes associated with metabolic and physiological changes allow *Arabidopsis thaliana* to respond to S limitation or restriction [4-13]. First, the limitation of S supply provokes a decrease in cysteine and an increase in O–acetylserine (OAS), its precursor. The accumulation of OAS and the decrease in GSH are then presumed to regulate the expression of numerous genes, such as the induction of genes implicated in S uptake, assimilation and redistribution, improving the acquisition and the utilisation of S for plant growth [14]. Nevertheless, as reported by Rouached *et al.*[15], these regulatory roles are questioned in the light of a number of experimental outcomes. Oilseed rape is also able to enhance its S remobilisation efficiency to sustain the S demand for growth under S restriction [3,16,17]. This is highly related to (i) the level of the SO_4_^2–^ pool previously stored in source leaves and (ii) the up–regulation of *BnSultr4;1* and *BnSultr4;2* expression [17], which are two genes encoding transporters that have been implicated in vacuolar efflux of SO_4_^2–^[18]. Other sulphate transporter genes in oilseed rape leaves and roots also respond positively to S limitation, leading to an increase in sulphate absorption and transport capacities at the whole plant level [16]. In spite of these processes, a lasting S limitation leads to an accumulation of amino acids, which is assumed to down–regulate nitrogen uptake and assimilation, while processes that increase the turnover of organic S compounds and stress defence responses are induced. Severe S limitation can ultimately result in a reduced growth, which is particularly associated with a reduced shoot:root ratio (for review see [19]).

Compared to the numerous results obtained through metabolomic and transcriptomic approaches, studies of proteomic modifications occurring in response to S restriction remain scarce in the *Brassicacea* family. However, this kind of approach has the advantage of integrating the regulation of gene expression, taking into account any post–transcriptional control. Indeed, transcriptome analysis is not sufficient for observing such regulation and does not completely predict the corresponding proteomic profile, especially in allopolyploid species such as oilseed rape. As recently reported by Marmagne *et al.*[20] in different neo synthesised oilseed rape lines, the majority of genes encoding proteins that exhibit additive gene expression are not expressed additively at the protein level. Such differences between transcription and protein expression could also occur in the case of S limitation, which could partially explain the observed temporal differences between metabolomic and transcriptomic responses [10]. Additionally, the decrease in cysteine and methionine contents from restriction of S could have an impact on the expression of essential proteins. The effect of S limitation on the proteome was mentioned in regard to *Arabidopsis* by Higashi *et al.*[21]. These authors have reported a significant disruption in the seed proteome in response to a S restriction, such as a reduction in the expression of proteins rich in S–amino acids (At2S3 and At12S3) that was not related to the accumulation of corresponding mRNAs. Therefore, a proteomic approach is particularly relevant in oilseed rape for the study of S limitation impacts on metabolic pathways. In order to address this question on a major oleaginous crop such as oilseed rape, our study aims to determine the leaf proteome modifications caused by a long–term S depletion occurring at the rosette stage (vegetative stage). This proteomic approach was combined with a physiological study to provide new insights about the plant response to S restriction.

## Results

### Impact of S limitation on physiological parameters

**Figure 1:**
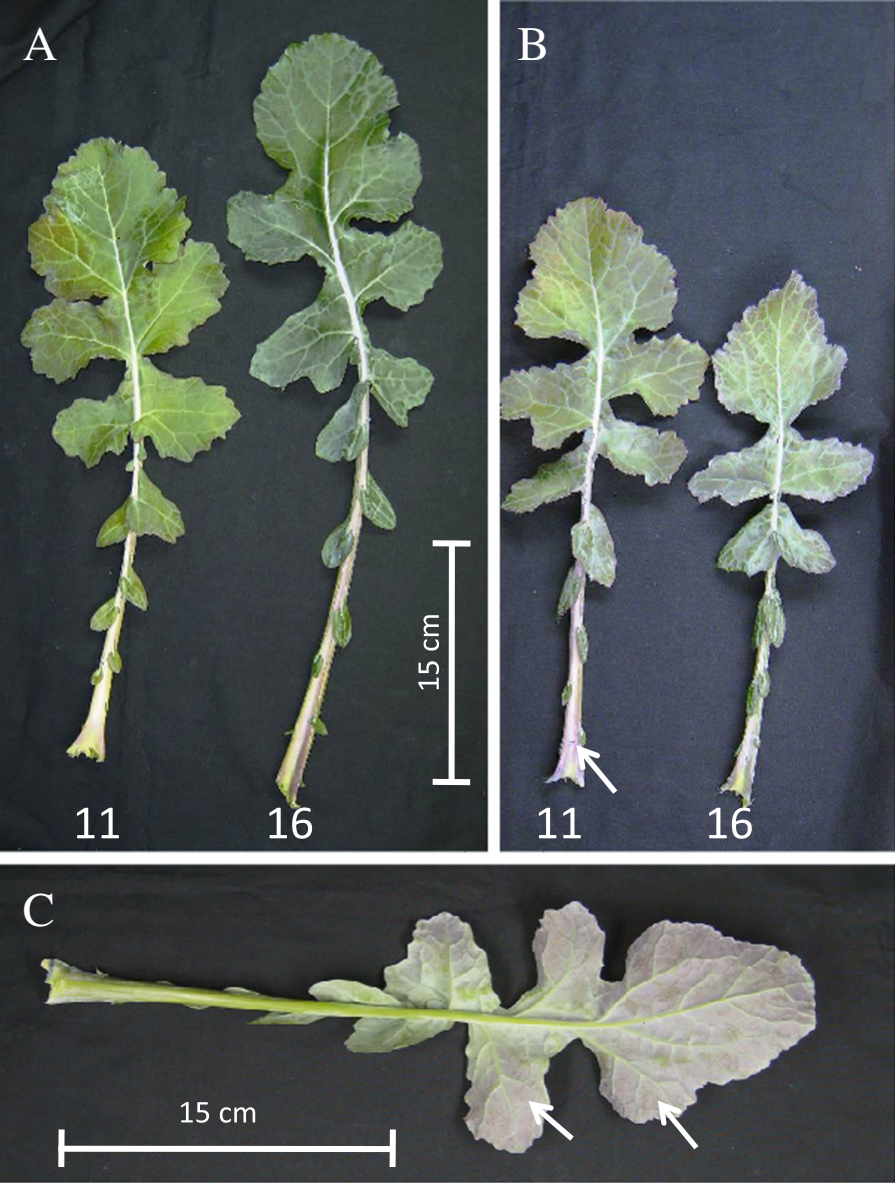
**Leaves #11 and 16 of a Control plant (panel A) and those subjected to an S restriction (panel B) over 35 d.** The abaxial face of leaf rank #16 of an S-restricted plant (Low S, **panel C**) and the petiole of leaf rank #11 (**panel B**) show a violet colour (indicated by white arrows).

At the rosette stage, the Low S treatment did not affect shoot and root growth significantly, compared to the Control (Table 1). However, a slight increase in the shoot:root ratio appeared after 35 d of S restriction (Low S) compared to Control plants. The growth of leaf #11, identified as a young leaf at the beginning of S treatment, and leaf #16, which appeared between 14 and 21 d after initiation of treatment, did not differ depending on the level of S supply (Table 1). Despite such lack of difference in growth, the length of petioles (Figure 1) as well as the biomass of petioles of younger leaves (i.e. above the leaf #16, Table 1) were significantly reduced by 35 d of Low S treatment (4.83 ± 0.83 g) compared to the Control (9.69 ± 1.63 g).

**Table 1:** Total shoot and root dry matter (DM), shoot:root ratio and DM of leaves #11 and 16 of plants subjected to Control and Low S treatments

		**Days of treatment**				
		**0**	**14**	**21**	**28**	**35**
**Shoot DM (g)**	**Control**	26.24 ± 2.09	32.86 ± 5.09	44.11 ± 5.20	62.05 ± 4.38	64.69 ± 4.33
	**Low S**	26.24 ± 2,09	34.02 ± 5.09	43.87 ± 5.20	55.29 ± 4.38	66.48 ± 4.33
**Root DM (g)**	**Control**	4.42 ± 0.94	7.38 ± 1.90	13.18 ± 5.02	16.06 ± 1.07	15.56 ± 2.11
	**Low S**	4.42 ± 0.94	11.10 ± 4.04	12.26 ± 3.35	19.20 ± 2.64	19.45 ± 3.22
**Shoot/Root ratio**	**Control**	6.73 ± 1.32	5.43 ± 1.53	5.11 ± 1.57	3.86 ± 0.02	4.34 ± 0.51
	**Low S**	6.73 ± 1.32	5.18 ± 2.10	4.67 ± 1.38	3.08 ± 0.52	3.72 ± 0.71
**DM of leaf # 11 (g)**	**Control**	1.74 ± 0.22	3.31 ± 0.86	3.53 ± 0.31	4.16 ± 0.41	3.11 ± 0.44
	**Low S**	1.74 ± 0.22	3.17 ± 0.44	3.18 ± 0.32	3.65 ± 0.13	3.81 ± 0.09
**DM of leaf # 16 (g)**	**Control**	-	-	2.29 ± 0.47	4.51 ± 0.32	4.07 ± 0.63
	**Low S**	-	-	1.58 ± 0.26	3.31 ± 0.83	3.51 ± 0.56

Data are means ± standard error (SE, *n*=4). None significant difference from the Control was observed.

**Figure 2:**
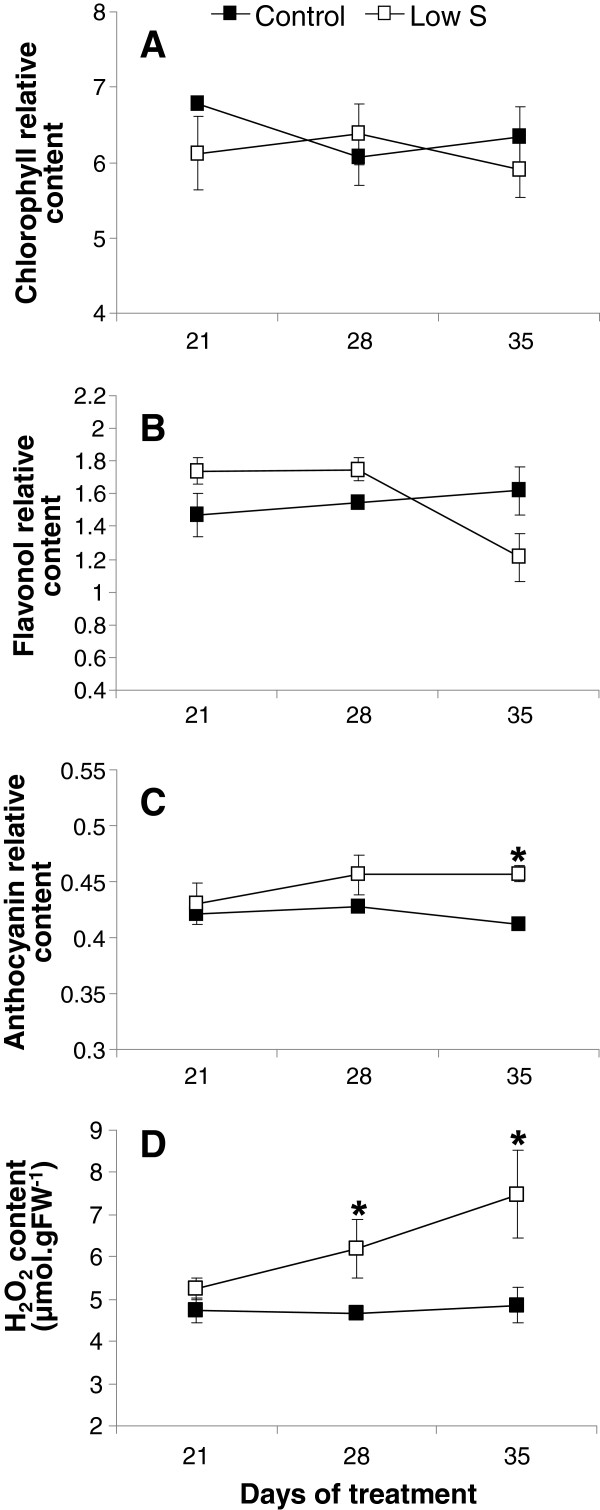
**Relative contents of chlorophylls (A), flavonols (B), anthocyanins (C) and H**_**2**_**O**_**2 **_**content (D) in leaf rank #16 of Control and S-restricted plants (Low S) after 21, 28 and 35 d of treatment.** Data are means ± SE (*n*=3). Vertical bars fit within the symbol if not visible. *: Significant differences from the Control value at p ≤ 0.05.

There were no significant differences in the chlorophyll and flavonol contents in leaves #11 (data not shown) and #16 (Figure 1A and 2B). However, after 35 d of S restriction, a significant increase in the relative anthocyanin content was observed in leaf #16 compared with Control (Figure 2C). In particular, this increase was also visible on the abaxial face of leaf #16 of Low S plants, which showed a violet colour that is indicative of anthocyanins at 35 d of S restriction (Figure 1C).

**Figure 3:**
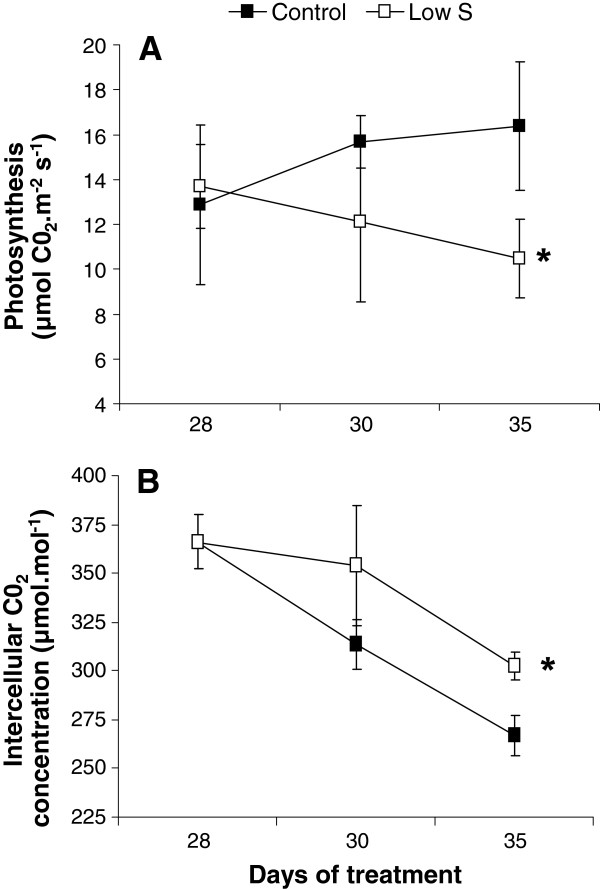
**Photosynthetic activity (A) and intercellular CO**_**2 **_**concentration (B) in leaf rank #16 of Control and S–restricted plants (Low S) after 28, 30 and 35 d of treatment.** Data are means ± SE (*n*=3). *: Significant differences from the Control value were at p ≤ 0.05.

**Figure 4:**
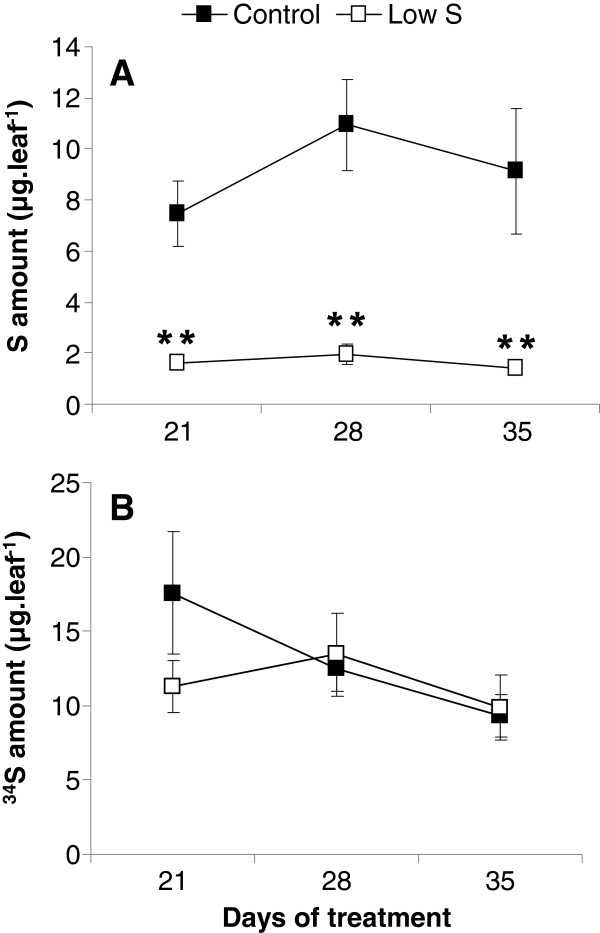
**Amounts of S (A) and **^**34**^**S (B) in leaf rank #16 of Control and S–restricted plants (Low S) after 21, 28 and 35 d of treatment.** Data are means ± SE (*n*=3). **: Significant differences from the Control value were at p ≤ 0.01.

A significantly lower photosynthetic activity (Figure 3A) and a higher intercellular CO_2_ concentration (Figure 3B) were also observed in leaf #16 of S restricted plants (Low S) compared to Control after 35 d. The S amounts of leaf #16 at 21, 28 and 35 d of treatment were significantly lower in Low S plants (Figure 4A), while the ^34^S quantities did not differ in this leaf between the two considered treatments (Figure 4B).

In order to evaluate the impact of S restriction on the occurrence of oxidative stress, the H_2_O_2_ content was determined in young leaf (Figure 2D). While the H_2_O_2_ content of young leaf at position #16 remained unchanged after 21 d, it was significantly higher after 28 d of S limitation compared to Control conditions. After 35 d of S treatment, H_2_O_2_ content in the young leaf of S-limited plants was 1.5-fold higher than in the young leaf of Control plants (Figure 2D).

### S restriction affects the young leaf proteome

**Figure 5:**
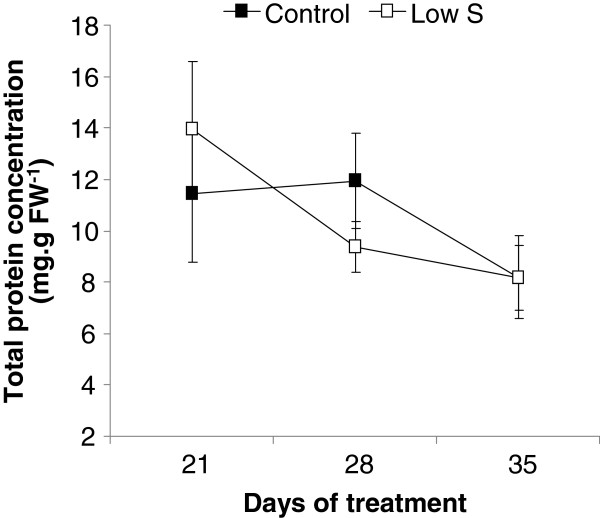
**Changes in amount of total proteins in leaf rank #16 of Control and S–restricted (Low S) plants after 21, 28 and 35 d of treatment.** Data are means ± SE (*n*=4). None significant difference from the Control was observed.

**Figure 6:**
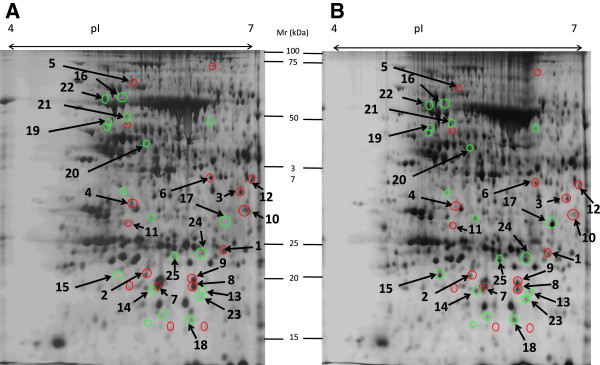
**Silver–stained two dimensional electrophoresis gels (2–DE) of total proteins from leaf rank #16 in Control (A) and S–restricted (B) plants after 35 d of treatment.** The spots circled in green and red are respectively induced and repressed in S–restricted plants compared with Control plants. The numbered spots were identified by LC–MS/MS and are listed in Tables 2 and 3. *Mr*: Molecular Weight; p*I*: isoelectric point.

The proteomic profiles of leaf #16 were compared after 35 d of treatment between Control and Low S plants. The total protein extracts showed no significant difference in protein content between these two treatments (Figure 5). Analysis of gels obtained after two–dimensional electrophoresis revealed that 36 protein spots were modulated in this leaf in response to S limitation compared to the Control (Figure 6). Beyond those spots, 19 and 17 spots were respectively induced and repressed by Low S treatment. LC–MS/MS enabled the identification of 25 spots, as shown in Tables 2 and 3.

**Table 2:** Proteins significantly repressed in leaf #16 after 35 d of sulphur restriction (Low S) identified by LC–MS/MS

**Spot No.**	**Normalized spot volume (Mean±SE x1E+06)**				**Fold change**	**Exp. pI / Mr**	**Theo. pI / Mr**	**PM**	**Score**	**SC (%)**	**Protein name / Species / NCBI Accession number**
	**Control**		**Low S**								
**1**	11.16	±1.07	7.85	±0.29	**−1.42**	6.5 / 24	8.5 / 59.1	4	29	7	Glutathione S-transferase / *Brassica oleracea* / gi|171921127
**2**	12.49	±0.39	9.55	±0.35	**−1.31**	5.6 / 21	5.8 / 26.3	6	159	11	Photosystem I light-harvesting chlorophyll a / b-binding protein / *Nicotiana tabacum* / gi|493723
**3**	14.93	±0.09	10.83	±1.04	**−1.38**	6.7 / 33	8.7 / 41.1	5	204	17	FNR2 (Ferredoxin-NADP(+)-Oxidoreductase 2) / *Arabidopsis thaliana* / gi|145323954
**4**	9.39	±0.63	5.21	±0.37	**−1.8**	5.5 / 31	5.8 / 36.6	3	188	10	THI1; protein homodimerization / *Arabidopsis thaliana* / gi|15239735
**5**	1.70	±0.06	1.31	±0.08	**−1.3**	5.5 / 65	5.3 / 55.2	8	346	25	Mitochondrial chaperonin (HSP60) / *Arabidopsis thaliana* / gi|2924773
**6**	7.51	±0.27	5.88	±0.35	**−1.28**	6.4 / 35	8.5 / 42.3	13	516	39	Chloroplast malate dehydrogenase / *Brassica rapa* subsp. Pekinensis / gi|207667274
**7**	14.45	±0.95	9.59	±1.02	**−1.51**	5.8 / 19	6.8 / 21.8	3	112	17	Germin-like protein / *Arabidopsis thaliana* / gi|1755154
**8**	19.15	±0.65	13.37	±1.45	**−1.43**	6.2 / 19	6.8 / 21.8	3	94	17	Germin-like protein / *Arabidopsis thaliana* / gi|1755154
**9**	23.59	±1.66	17.53	±1.05	**−1.35**	6.2 / 20	6.8 / 21.8	3	102	17	Germin-like protein / *Arabidopsis thaliana* / gi|1755154
**10**	28.44	±0.85	19.68	±2.49	**−1.45**	6.8 / 30	6.2 / 27.5	14	619	55	Chain B, The Transient Complex Of Poplar Plastocyanin With Turnip Cytochrome F Determined With Paramagnetic Nmr / *Brassica rapa* / gi|67463833
**11**	2.90	±0.20	2.14	±0.17	**−1.36**	5.5 / 29	5.3 / 26.3	4	192	13	AT2G37660 / *Arabidopsis thaliana* / gi|227204455
**12**	9.24	±1.30	5.96	±0.37	**−1.55**	6.8 / 35	8.5 / 35.8	7	415	31	Mitochondrial malate dehydrogenase (NAD) / *Arabidopsis thaliana* / gi|18404382

Significant ANOVA was followed by a Mann–Whitney test (p≤0.05) carried out on the leaf–normalised spot volumes (*n*=3). Experimental and theoretical p*I* / *M*_*r*_, the number of LC–MS/MS matched peptides (PM), the SCORE and the percentage of sequence coverage (SC) obtained are also indicated. The assigned best–matched protein is listed with the organism in which it was identified and its GenBank protein accession number.

**Table 3:** Proteins significantly induced in leaf #16 after 35 d of sulphur restriction (Low S) identified by LC–MS/MS

**Spot No.**	**Normalized spot volume (Mean±SE x1E+06)**				**Fold change**	**Exp. pI / Mr**	**Theo. pI / Mr**	**PM**	**Score**	**SC (%)**	**Protein name / Species / NCBI Accession number**
	**Control**		**Low S**								
**13**	1.08	±0.18	12.47	±3.78	**11.51**	6.3 / 19	8.4 / 22.7	2	60	8	Water-soluble chlorophyll binding protein / *Brassica oleracea* var. acephala / gi|27530883
**14**	2.61	±0.40	13.75	±2.89	**5.27**	5.7 / 19	7.8 / 22.7	3	132	16	Water-soluble chlorophyll binding protein / *Brassica oleracea* var. acephala / gi|27530881
**15**	3.83	±1.88	11.77	±1.55	**3.08**	5.3 / 20	5.1 / 23.3	14	368	44	Trypsin inhibitor propeptide / *Brassica oleracea* / gi|841208
**16**	1.07	±0.09	3.11	±0.84	**2.92**	5.4 / 59	5.1 / 55.3	9	409	18	ATP synthase CF1 alpha subunit / *Brassica napus* / gi|262400756
**17**	2.97	±0.43	23.36	±1.06	**7.86**	6.6 / 29	6.5 / 28.8	7	175	15	BCA3 (BETA CARBONIC ANHYDRASE 4); carbonate dehydratase / zinc ion binding / *Arabidopsis thaliana* / gi|15220853
**18**	6.53	±0.32	9.69	±0.63	**1.49**	6.1 / 17	6.7 / 21.8	3	75	18	Cu-Zn Superoxide dismutase / *Arabidopsis thaliana* / gi|3273753
**19**	0.97	±0.15	2.22	±0.25	**2.29**	5.2 / 49	5.4 / 20.6	6	271	35	Putative myrosinase-binding protein 3 / *Brassica rapa* subsp. Pekinensis / gi|33285912
**20**	1.19	±0.13	2.73	±0.30	**2.3**	5.7 / 43	6.3 / 40.9	4	101	8	12-oxophytodienoate reductase / *Arabidopsis thaliana* / gi|2765083
**21**	2.82	±0.29	5.24	±0.70	**1.86**	5.4 / 51	7.6 / 48.7	2	77	5	Aminotransferase class I and II family protein / *Arabidopsis thaliana* / gi|15217440
**22**	8.10	±0.57	10.68	±0.44	**1.32**	5.2 / 57	5.0 / 54.7	21	954	50	Nucleotide-binding subunit of vacuolar ATPase / *Arabidopsis thaliana* / gi|166627
**23**	1.66	±0.25	4.81	±0.52	**2.89**	6.3 / 19	9.0 / 21.2	2	91	10	Unknown protein / *Arabidopsis thaliana* / gi|7658343
**24**	6.42	±0.36	14.89	±1.60	**2.32**	6.3 / 24	5.8 / 21.6	4	145	19	Unknown protein / *Populus trichocarpa* / gi|118485421
**25**	2.77	±0.22	8.29	±1.21	**2.99**	5.9 / 24	5.8 / 21.6	3	118	19	Unknown protein / *Populus trichocarpa* / gi|118485421

Significant ANOVA was followed by a Mann–Whitney test (p≤0.05) carried out on the leaf–normalised spot volumes (*n*=3). Experimental and theoretical p*I* / *M*_*r*_, the number of LC–MS/MS matched peptides, the SCORE and the percentage of sequence coverage (SC) obtained are also indicated. The assigned best–matched protein is listed with the organism in which it was identified and its GenBank protein accession number.

Among the 17 proteins that were repressed in Low S conditions (Table 2), several chloroplastic proteins were characterized: a photosystem I chlorophyll a/b binding protein (spot No. 2); a protein showing similarity with ferredoxin–NADP reductase (FNR, spot No. 3); and a chloroplastic malate dehydrogenase (MDH; spot No. 6) that could be involved in the “malate valve”. The “malate valve” catalyses the export of malate from the chloroplast when the NADPH to NADP^+^ ratio is high [22]. THI1 (spot No. 4), a protein located in the chloroplast and mitochondrion that is involved in thiamine synthesis, was also repressed. A mitochondrial chaperonin, Heat Shock Protein (HSP; spot No. 5), and a glutathione S–transferase (spot No. 1), an enzyme especially involved in detoxification of xenobiotics, were also negatively affected. Spots 7, 8 and 9, similarly repressed by the S restriction treatment, were identified as germin–like proteins, which may present an oxalate oxidase activity [23].

Spots 13 and 14, strongly induced in our study (11.5 and 5.3 fold respectively) correspond to chloroplastic Water Soluble Chlorophyll binding Proteins (WSCPs) in *Brassica oleracea*, which present a dual function of protection of chlorophyll against reactive oxygen species (ROS) and protease inhibitor activity [24]. A trypsin inhibitor propeptide (spot No. 15), was also significantly induced in leaf #16 after 35 d of S restriction (Low S), compared to Control. The ATP synthase F1–subunit (spot No. 16), responsible for the ATP synthesis that occurs during the light phase of photosynthesis, was also induced. A strong accumulation of a β–Carbonic anhydrase was also observed (factor 7.9; spot No. 17). This enzyme, that reversibly catalyses CO_2_ hydration into carbonate (H_2_CO_3_), is involved in various metabolic processes [25]. Similarly, a protein associated with the chloroplastic Cu-Zn superoxide dismutase (Cu–Zn SOD, spot No. 18) encoded by the *CSD2* gene in *A. thaliana*[26] was induced in leaf #16 after 35 d of S restriction. This protein is well known to be involved in defence against oxidative stress. A spot identified as a putative myrosinase–binding protein from *Brassica rapa* (spot No. 19) was also induced under S restriction. The enzymes 12–oxophitodienoate reductase (spot No. 20) and 1–aminocyclopropane–1–carboxylate synthase (ACC synthase, spot No. 21), which catalyse jasmonate and ethylene synthesis respectively [27,28] were similarly induced in leaf #16 after 35 d of S restriction. Finally, a slight induction was observed for a vacuolar ATPase subunit (spot No. 22), a tonoplastic protein involved in active transport in vacuoles [29].

## Discussion

### S limitation at the rosette stage does not change the total shoot biomass but inhibits growth and photosynthesis of young leaves

As previously described in *Brassica olearacea*[30,31] and oilseed rape [17,32], S restriction applied at the rosette stage over 35 d does not result in a significant inhibition of the total shoot growth (Table 1). However, these results contrast with two studies showing a growth reduction after a shorter period of S depletion in oilseed rape [16,33]. In these cases, the chlorophyll content of young leaves was also affected by the S restriction, a symptom that was not found in our study. Our finding suggests that during the 55 d preceding the sulphate limitation, plants have sufficiently absorbed and stored S for sustainable growth and maintenance of the various physiological processes measured during the following 35 d of treatment. It thus appears that at the rosette stage, S limitation has varying effects on oilseed rape, depending on the initial level of S storage.

Nevertheless, in our experiment, a significant reduction in biomass and length of petioles in the younger leaves (Figure 1B) was observed after 35 d of Low S treatment compared to the Control. If this reduction in petiole length is confirmed in further experiments, it could be possible to use this morphological trait as an indicator of S deficiency during the early vegetative stage of oilseed rape development. The total S amount in young leaf #16 of Low S plants was significantly lower than Control (Figure 4A). This indicates that S limitation has a negative impact on young leaf metabolism. Indeed, this is highlighted by a significant increase in anthocyanin content (Figures 1 and 2C), a decrease in photosynthetic activity (Figure 3A), and is associated with a higher intercellular CO_2_ concentration (Figure 3B). Proteome analysis performed on the young leaf #16 provides evidence that these physiological alterations were related to modulations of protein expression leading to metabolic changes that occurred in response to 35 d of S restriction.

### Proteins associated with S metabolism and remobilisation of S compounds are specifically modulated by S restriction

Among the physiological responses that may contribute to compensating for low S nutrition, the remobilisation of S reserves is a major process. Using ^34^S labelling, it appeared that Low S plants are able to maintain the amount of ^34^S in young leaves at a relatively stable level compared to the Control (Figure 4B). In contrast, leaf #16 of Control plants undergoes ^34^S isotope dilution associated with the chase–period (Figure 4B), attesting that unlabelled S is absorbed and transported to this young leaf. As previously reported [16,17], the redistribution of S in response to S limitation can be achieved by a strong remobilisation of previously stored sulphate, through a tissue–specific induction of genes encoding the sulphate transporters, Sultr4;1 and 4;2, which are involved in the efflux of sulphate accumulated in the vacuolar compartment. The proteomics approaches (Table 3) revealed the induction of a vacuolar ATPase subunit, which could be implicated in S remobilisation processes through the maintenance of an efficient sulphate efflux from the vacuole, so as to sustain growth [17,29].

Our proteomic analysis does not reveal modulation of proteins associated with primary S metabolism, probably due to the fact that the proteomic study was performed after 35 d of S limitation. However, some proteins implied in secondary S metabolism are affected by S limitation. The putative myrosinase–binding protein is induced by S restriction. Because of its potential involvement in the regulation of myrosinase activity, this result suggests that glucosinolates can be used as a sulphate source in cases of severe S limitation. This finding is consistent with transcriptomic data and metabolome analysis in *Arabidopsis thaliana* that reveal an induction of myrosinase binding protein gene induction [9] and showed a decrease in the accumulation of glucosinolates in S restricted plants [6]. Also, the repression of THI1, involved in thiamine biosynthesis, may lead to a preferential allocation of cysteine for GSH and protein synthesis, since thiamine is produced from glyceraldehyde–3–phosphate and cysteine, two molecules whose levels are affected by the S limitation [11,13]. Similarly, after 35 d of S restriction, the repression of glutathione S–transferase (Table 2), also shown at the transcriptomic level in *Arabidopsis thaliana*[34], could reduce the xenobiotic detoxification capacity in the young leaf, may allow regulation of GSH utilisation for other purposes. These proteomic changes associated with the lower S content of leaf #16 observed in cases of S restriction clearly indicate a lack of S for the proper metabolism of this leaf.

### Proteins involved in C metabolism and processes related to energy production are impacted by S restriction

In young leaves, C metabolism appears to be affected by 35 d of S limitation, and particularly photosynthetic metabolism (Figure 3A), which leads to a C fixation decline and a higher intercellular CO_2_ concentration (Figure 3B). The proteomics approaches performed in the present study (Tables 2 and 3) helped to understand how the S restriction interacts with C metabolism by determining the impact of S limitation on the light and dark reactions of photosynthesis.

**Figure 7:**
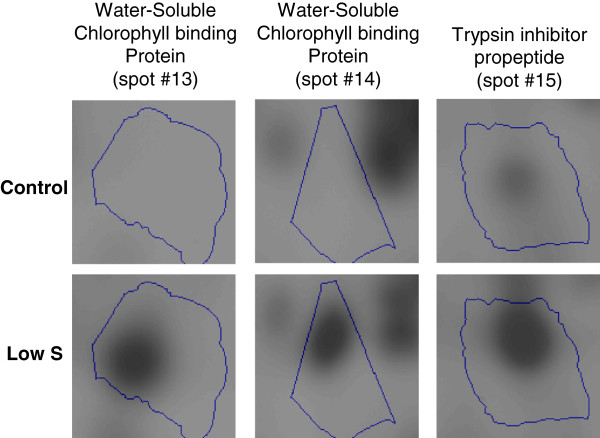
**Changes in abundance of protein spots #13 (WSCP), 14 (WSCP), and 15 (Trypsin inhibitor propeptide) in leaf rank #16 of Control and S–restricted plants.** Details about protein identification are given in Tables 2 and 3. WSCP: Water-Soluble Chlorophyll binding Protein.

The reduction of a putative Chl*a*/*b* binding protein could cause an inhibition of photosynthetic activity in young leaves, since this protein belongs to the photosystem I Light Harvesting Complex (LHCI) and is involved in chlorophyll protection against degradation. In *Arabidopsis thaliana,* numerous genes encoding for this protein were also repressed in response to S depletion [4,9]. However, the impact of a lower accumulation of this Chl*a*/*b* binding protein would be minimal since the S restriction applied in our experiment did not result in altering the chlorophyll level of the young leaves studied. Spots no. 13 and 14 (Figure 7), identified as Water Soluble Chlorophyll binding Protein (WSCP), correspond to serine protease inhibitor that can bind to chlorophyll. The accumulation of WSCPs such as WSCP1, WSCP2 and BnD22 is also observed in young leaves of oilseed rape subjected to a nitrogen starvation (0 mM NO_3_^–^), in comparison with well–fed oilseed rape (3 mM NO_3_^–^) [24]. WSCPs, that are specific of *Brassicacea,* may also be involved in chlorophyll protection against ROS and in the maintenance of protein content [24,35-37]. Interestingly, proteomics approaches revealed the induction of Trypsin inhibitor propeptide (spot no. 15, Figure 7), which is able to inhibit proteases, binding with them in their active site. Therefore, the strong accumulation of WSCPs and Trypsin inhibitor propeptide could be involved in maintaining the protein content and chlorophyll level observed in leaf #16 under low S nutrition.

In contrast, two proteins that belong to the electron transfer chain in the thylakoidal membrane were repressed: plastocyanin (PC) and ferredoxin-NADP reductase (FNR). Because these two proteins act in the final stages of electron transfer during the light phase of photosynthesis and FNR catalyses the production of NADPH+H^+^ required for CO_2_ assimilation, it could be hypothesized that the first physiological symptoms of S limitation result in an alteration of the coupling between the light and dark phases of photosynthesis leading to a depletion of C assimilation by the limitation of NADPH+H^+^ availability. Indeed, sulphate restriction is known to affect C assimilation leading to a reduction in photosynthetic activity and a distortion of glycolytic flux, which can be assumed as a repercussion of amino acid accumulation, itself resulting from a reduction of S assimilation into cysteine [11,38]. These changes to proteins associated with C metabolism observed in our study may lead to the accumulation of intercellular CO_2_ (Figure 3B), which may finally result in the inhibition of growth in young leaves.

The strong accumulation of a β–carbonic anhydrase is also indicative of a C metabolism disruption in this leaf in response to 35 d of S restriction. This enzyme, which catalyses the reversible hydration of CO_2_ to form HCO_3_^–^ and H^+^, is directly involved in the CO_2_ metabolism associated with the Calvin cycle [25]. In *Arabidopsis thaliana*, S limitation also results in the induction of the gene encoding this protein in leaves [8]. The accumulation of this protein would allow a better solubilisation of CO_2_ at the cellular level and could therefore promote photosynthetic processes at the leaf level. It may also modify the pH of the intracellular medium, which could impact on numerous protein activities. Therefore, it can be assumed that the reduction in photosynthetic activity and the accumulation of CO_2_ at the intercellular level are not directly caused by the reduction in the cysteine content or OAS accumulation, but could be related closely to C metabolism disturbances.

Proteomic analysis also shows an induction of proteins implicated in the maintenance of energy production in young leaves subjected to Low S treatment such as the catalytic α–subunit of chloroplastic ATP synthase. The induction of this enzyme, involved in ATP synthesis at the end of the electron transport chain in the thylakoidal membrane, suggests that the production of ATP in chloroplasts is favoured in the case of S limitation. This hypothesis is supported by the induction of proteins (WSCPs, Trypsin inhibitor propeptide) involved in the maintenance of reactions of the light phase of photosynthesis, providing the proton gradient required for ATP synthesis.

Although the repression of the thiazole biosynthetic enzyme (THI1) may lead to a preferential allocation of cysteine, it could have a negative impact on carbohydrate metabolism, whether on glycolysis/neoglucogenesis or on chlorophyll synthesis. Thiamine, an S containing molecule, is the precursor of thiamine pyrophosphate, an essential co–enzyme for the activity of key enzymes involved in C metabolism such as pyruvate carboxylase, pyruvate oxidase or transketolase [39]. The repression of this enzyme could also cause chlorosis if S restriction is extended beyond 35 d. Indeed, in *Arabidopsis thaliana*, *AtTHI1* mutants result in a significant decrease in the chlorophyll level leading to photobleaching [40]. THI1 repression, similar to HSP60, is also a sign of mitochondrial stress caused by S limitation since this protein is also involved in mitochondrial DNA damage tolerance [41].

Proteomic analysis has also revealed the repression of a chloroplast malate dehydrogenase (MDH) suggesting a relative reduction in malate export [42]. Additionally, the repression of a mitochondrial MDH that uses NADH to reduce oxaloacetate to malate, which is then exported to the cytosol, also suggests an alteration of energy production through the Krebs cycle. This protein, highly regulated in a post–translational manner, is involved in the partitioning of C and energy, and here could be partly responsible for the reduction in net CO_2_ consumption, as suggested by Tomaz *et al.*[43]. These chloroplastic and mitochondrial MDH repressions could also be implied in the malate accumulation observed in oilseed rape in the case of S deficiency [44]. Indeed, with respect to sulphate decreases, malate may be transported into the vacuole where it could act as a counter ion for cations as assumed by Blake–Kalff *et al.*[44].

### S restriction provokes physiological and proteomic changes related to oxidative stress in young leaves

In accordance with the *Arabidopsis thaliana* transcriptome responses to S restriction [4,6,8,9], our proteomic study showed an accumulation of 12–oxophytodienoate reductase, which catalyses the last step of jasmonic acid biosynthesis. Jasmonic acid could have a positive effect on S metabolism in cases of S limitation through the induction of many genes such as *APS1* and *APS2* coding for ATP sulfurylase, *SAT3* coding for serine acetyltransferase [45]. Interestingly, among the genes induced by jasmonic acid, there is the myrosinase binding-protein [9]. Then, it could be postulated that the induction of this protein observed in our study is related to jasmonic acid accumulation in case of S limitation. Indeed, several S compounds play a role in plant stress tolerance [46], and jasmonic acid is known to participate in the transduction of stress responses [47]. This phytohormone could result ultimately in cell death, particularly *via* the induction of an oxidative stress. In response to S restriction, analysis of the leaf proteome also reveals the induction of ACC synthase, an enzyme implicated in the biosynthesis pathway of ethylene. Like jasmonic acid, ethylene plays a potential role in the stimulation of ROS accumulation, which acts as a signalling molecule for inducing plant responses against biotic and abiotic stresses [48].

The accumulation of the chloroplastic Cu–Zn SOD, associated with O_2_^.-^ detoxification, also indicates that S limitation causes an oxidative stress in young leaves, as previously observed under S deficiency in mulberry plants [49]. The repression of protein spots showing similarity to germin–like proteins, which could have an oxalate oxidase activity that generates H_2_O_2_ and CO_2_[23], may thus be involved in reducing oxidative stress. Furthermore, the strong accumulation of the β–carbonic anhydrase also suggests a possible antioxidant function of this enzyme as mentioned by Slaymaker *et al.*[50]. All these results together reinforce the idea that S restriction leads to an oxidative stress that seems to be actively attenuated in young leaves by the modulation of various proteins involved in resistance to oxidative stress. However, our findings show an increase in H_2_O_2_ content after 28 and 35 d of S restriction, demonstrating that these defence mechanisms do not appear entirely effective.

## Conclusions

**Figure 8:**
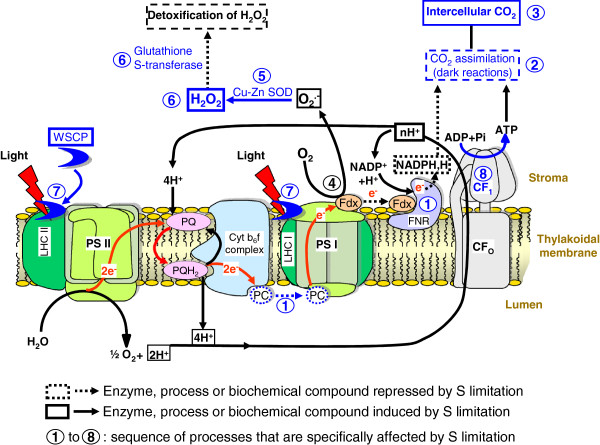
**Putative sequence of events provoked by a 35 d S limitation on the electron transfer chain and ATP synthase in a young leaf chloroplast.** Proteines processes (1 to 8) or biochemical compounds that are effectively repressed or induced by S restriction are indicated respectively in doted or plain blue lines, while those that are postulated to be repressed or induced are in black lines. In Control, the electron transfer chain produced NADPH required for CO_2_ assimilation. After 35 d under Low S conditions, we observed: **1**, a repression of ferredoxin-NADP reductase (FNR) and plastocyanin (PC) (Table 2) which could cause a perturbation of the electron transfer and a lower production of NADPH+H^+^; **2**, the decline in CO_2_ assimilation (Figure 3B), probably linked to NADPH+H^+^ depletion; **3**, an intercellular CO_2_ accumulation (Figure 3A) concomitant with the photosynthesis reduction; **4**, the lower abundance of FNR may also result in the transfer of electrons to O_2_ by Ferredoxin (Fdx), producing O_2_^.-^; **5**, a higher abundance of Cu-Zn Superoxide Dismutase (SOD) (Table 3), which suggests that the of O_2_^.-^detoxification into H_2_O_2_ could be increased; **6**, an accumulation of H_2_O_2_ (Figure 2D) probably due to ineffective detoxification process such as the repression of glutathione S-transferase, which leads to an oxidative stress; **7**, an accumulation of Water Soluble Chlorophyll binding Protein (WSCP) while the chlorophyll content is maintained (Figure 2A), may signify that chlorophylls are protected against oxidative stress and that the photosystems remain efficient; **8**, an accumulation of ATP synthase F1 complex, which in association with the H^+^ accumulation in the lumen due to proper functioning of photosystems and electron chains, suggests that ATP production is favoured. CF_O_: membrane-embedded subunit of ATP synthase; CF_1_: catalytic subunit of ATP synthase; Cyt b_6_f: cytochrome b_6_f complex; LHC: Light Harvesting Complex ; PQ: Plastoquinone.

A relatively long period (35 d) of S limitation affects C metabolism in the young leaves of oilseed rape, and in particular the photosynthetic activity through the repression of dark reactions, as evidenced by the reduction of CO_2_ assimilation and the accumulation of intercellular CO_2_. A general scheme for summarizing the cascade of events that explain the impact of S limitation on photosynthesis was proposed in Figure 8. The reduction in CO_2_ assimilation in young leaf is not due to a decrease in chlorophylls, which remains stable, but it is probably related to an alteration of the final stages of electron transfer and a limitation of NADPH+H^+^ due to the repression of PC and FNR. The accumulation of H_2_O_2_ and anthocyanins indicated that S limitation also provokes an oxidative stress in young leaves. This could be explained by (i) the repression of FNR that might amplify the formation of ROS by the Mehler reaction, resulting in the transfer of electrons to O_2_ by ferredoxin, producing O_2_^.-^, (ii) the higher abundance of Cu-Zn SOD, which detoxifies O_2_^.-^ into H_2_O_2_, and (iii) the up-regulation of enzymes involved in the synthesis of jasmonic acid (12–oxophytodienoate reductase) and ethylene (ACC synthase), two phytohormones potentially involved in the induction of oxidative stress. Simultaneously, (i) chlorophylls and protein contents remained stable, (ii) WSCPs which are involved in the protection of chlorophyll against photooxidation, were induced, and (iii) ATP synthase F1 complex were accumulated. This suggests that, despite the occurrence of an oxidative stress, the capacity of leaves to absorb photosynthetic active radiations by the photosystems and ATP production remain efficient for as long as possible. However, these protection mechanisms against ROS damage via the regulation of ROS production and detoxification are not fully effective to enable tolerance to a long period of S limitation.

Our proteomic study does not reveal inductions of well known biomolecular markers of S deficiency that were identified in *Arabidopsis thaliana* such as isoflavonoïde reductase, involved in anthocyanins synthesis, or primary S metabolism enzymes [5,9]. Then, it could be interesting to perform a kinetic study of changes in leaf proteome to determine the first events associated with S limitation, and verify if these typical marker genes are also detectable using 2-DE in *Brassica napus*.

## Methods

### Plant material, experimental treatments and tissue sampling

Seeds of *Brassica napus* L. (cv. Capitol) were sterilised by exposure to 80% ethanol for 30 s followed by treatment with 1% sodium hypochlorite for 10 min under agitation and then washed thoroughly with demineralised water. Then seeds were germinated on perlite soaked with ¼ Hoagland nutrient solution consisting of 1.25 mM Ca(NO_3_)_2_,4H_2_O, 1.25 mM KNO_3_, 0.5 mM MgSO_4_, 0.25 mM KH_2_PO_4_, 0.2 mM Fe–Na EDTA, 14 μM H_3_BO_3_, 5 μM MnSO_4_, 3 μM ZnSO_4_, 0.7 μM (NH_4_)_6_Mo_7_O_24_, 0.7 μM CuSO_4_, 0.1 μM CoCl_2_, which included sulphate-labelled with ^34^S isotope at 1% atom excess in K_2_SO_4_ form as previously described by Dubousset *et al.*[17]. Five–day–(d)–old seedlings were then transplanted at 18 plants per tray into a hydroponic system supplied with 20 L of ¼ Hoagland nutrient solution containing ^34^SO_4_^2–^, constantly aerated and renewed every 7 d. After 55 d in these conditions, plants were grown in individual containers filled with 4 L of nutrient solution, and then the ^34^SO_4_^2–^ labelling was stopped, giving way to a chase period of 35 d where two levels of S supply were applied: 500 μM for Control and 8.7 μM MgSO_4_ for S limited plants (Low S). The lack of Mg was compensated with addition of MgCl_2_. These nutrient solutions were renewed every 7 d. During the whole experiment, plants were illuminated by natural light, supplied with Philips® Green Power lamps (400 μmol.m^–2^.s^–1^ photosynthetically active radiation in the canopy) for 16 h per day, and subjected to a thermoperiod of 20°C (day) and 15°C (night). Leaves were numbered in order of their appearance and therefore according to their nodal position.

Four plants of each treatment (Control, Low S) were harvested after 0, 14, 21, 28 and 35 d of treatment. Leaf fresh mass and leaf area were measured. An aliquot of each leaf was freeze–dried to determine the dry matter, and samples were ground into a fine powder to determine their S and ^34^S content. Aliquots of about 200 mg of fresh matter of each organ were immediately frozen after harvest in liquid nitrogen and stored at -80°C to extract total proteins.

### Measurement of physiological parameters during application of treatments

All measurements were made on three or four plants for each treatment (Control and Low S) to allow statistical analysis, and two or three technical replicates were conducted. The levels of chlorophylls, anthocyanins and flavonols in leaves were measured each week from the beginning of the S treatment in Control and Low S plants with an optical sensor system (Multiplex ®, Force A, Orsay, France). Gas exchanges for the photosynthetic parameter measurements were performed during the last days of treatment, between 9:00 and 12:00, with a portable LI–6400 system for measuring gas exchange (LI–COR, Inc., Lincoln, NE, USA) in leaves from rank #7 (mature leaf at the beginning of treatment), rank #11 (young leaf at the beginning of treatment) and rank #16 (young leaf emerged during treatment). Net photosynthesis and intercellular CO_2_ concentration were determined in these leaves at 20°C, at approximately 400 ppm CO_2_ and with a photosynthetically active photon flux of 1000 μmol.m^–2^.s^–1^.

### S and ^34^S analysis

Freeze–dried samples were ground to a fine powder, weighed, and placed into tin capsules. S content was determined with an elemental analyser (EA3000, EuroVector, Milan, Italy) connected to a continuous flow isotope mass spectrometer (IRMS, Isoprime, GV Instruments, Manchester, UK). The IRMS analysis also provided the changes in the relative amount of ^34^S in excess in each sample derived from the tracer fed to the plant as described previously by Dubousset *et al.*[3].

### Determination of H_2_O_2_ content

The H_2_O_2_ content was determined as described by Lee *et al.*[51]. About 500 mg FW of leaf samples were homogenized with 1.5 mL of 50 mM phosphate buffer (pH 6.8) and then centrifuged at 6000 *g* for 25 min. The resulting supernatant was then mixed with 1 mL of 0.1% titanium chloride in 20% (v/v) H_2_SO_4_ and centrifuged at 6000 *g* for 15 min. The absorbance of the resulting supernatant was immediately read at 410 nm and H_2_O_2_ concentration was calculated using a linear calibration curve of H_2_O_2_ solutions ranging from 0 to 10 mM.

### Extraction and determination of total proteins

Two hundred milligrams of fresh leaf samples were ground to a fine powder in liquid nitrogen in the presence of 50 mg of poly(vinylpolypyrrolidone) (PVPP). The addition of PVPP is used to fix plant polyphenols that might interfere with the quantification of proteins or during separation of proteins by electrophoresis. The ground material was dissolved in 1.75 mL of TCA/acetone solution (10% TCA (w/v) prepared in acetone). After centrifugation (3 min, 16000 *g*, 4°C), the protein pellet was purified according to the protocol adapted from Wang *et al.*[52]. The protein pellet obtained after precipitation with TCA/acetone was resuspended in 1.75 mL of 0.1 M ammonium acetate dissolved in 80% methanol. After homogenisation and centrifugation (16000 *g*, 3 min, 4°C), the pellet was washed with 1.75 mL of 80% acetone and centrifuged again (16000 *g*, 3 min, 4°C). The supernatant was removed and the pellet was dried under vacuum (Speedvac Concentrator 5301, Eppendorf, France) for 5 min at 50°C and then resuspended in 0.8 mL phenol at pH 7.9 and in 0.8 mL of dense SDS buffer (30% sucrose, 2% SDS, 0.1 M Tris–HCl, pH 8.0, 0.5% 2–mercaptoethanol). After 5 minutes incubation at 4°C and centrifugation (16000 *g*, 3 min, 4°C), the phenol phase was transferred to a new tube and supplemented with 1.75 mL of 0.1 M ammonium acetate and stored at –20°C overnight. Afterwards, ammonium acetate was used to precipitate proteins to enable their collection by centrifugation (16000 *g*, 5 min, 4°C). The protein pellet was then washed with 1.75 mL of 100% methanol and again with 1.75 mL of 80% acetone. Residual acetone was removed by vacuum evaporation over a few minutes. The pellet was resuspended in 400 μL of rehydration R2D2 buffer [5 M urea, 2 M thiourea, 2% CHAPS, 2% N–decyl–N,N–dimethyl–3–ammonio–1–propanesulfonate, 20 mM dithiothreitol, 5 mM Tris (2–carboxy– ethyl) phosphine, 0.5% IPG buffer (GE Healthcare, Saclay, France), pH 4 to 7; [53]]. The total protein concentration was determined by the method of Bradford [54] using bovine serum albumin as standard.

### Two–dimensional electrophoresis (2–DE) and image analysis

For 2–DE, we followed the protocol detailed by Desclos *et al.*[24]. Gels were stained using the silver–staining procedure described by Blum *et al.*[55] and scanned with the ProXPRESS 2D proteomic imaging system (Perkin–Elmer, Courtaboeuf, France) before image analysis. Images of the 2–DE gels were analysed using the Progenesis SameSpots software v3.0 (Nonlinear Dynamics, Newcastle upon Tyne, UK) according to the manufacturer’s protocol. Gels from four independent biological replicates were used. Spot detection, warping, and matching were performed automatically by the software and manually validated. Artefacts due to non–specific silver nitrate staining or spots that could not be confidently verified as true matches were disregarded rather than manually edited, and misalignments were corrected by manual warping when appropriate. *M*_r_ and p*I* were calculated using Samespots software calibrated with commercial molecular mass standards (prestained precision protein standards; Bio–Rad, Marne–la–Coquette, France) run in a separate marker lane on 2–DE gel.

### Protein Identification by ESI LC–MS/MS

Spots of interest were excised and washed several times with water and dried for a few minutes. Trypsin digestion was performed overnight with a dedicated automated system (MultiPROBE II, Perkin-Elmer). The gel fragments were subsequently incubated twice for 15 min in a 0.1% CH_3_CN solution in water to allow extraction of peptides from the gel pieces. Peptide extracts were then dried and dissolved in starting buffer for chromatographic elution, consisting of 3% CH_3_CN and 0.1% HCOOH in water. Peptides were enriched and separated using lab–on–a–chip technology (Agilent, Massy, France) and fragmented using an on–line XCT mass spectrometer (Agilent). The ESI LC–MS/MS data were converted into DTA–format files that were further searched for proteins with MASCOT Daemon (Matrix Science, [56]). For protein identification, two strategies were employed to mine the maximum information. Measured peptides were searched in the NCBInr–protein sequence database, viridiplantae (green plants), and in the Brassica EST database (Brassica Genome Gateway 2007, [57]). Proteins with two or more unique peptides matching the protein sequence with a score >53 defined by MASCOT, were considered as a positive identification. The spectra of each peptide were verified manually.

### Statistics

The variability of the results is expressed by the average values for all biological replicates (*n* = 3 or 4) ± standard error (SE). For each harvest date, the effects of Low S treatments compared to the Control were subjected to statistical analysis using Microsoft® Excel 2008/XLSTAT©-Pro (Version 7.2, 2003, Addinsoft, Inc., Brooklyn, NY, USA). With a statistical significance postulated at p<0.05, the Wilcoxon test was chosen to compare physiological parameters between treatments, whereas the Mann–Whitney test was done to compare S, ^34^S, H_2_O_2_ and protein expressions between Low S and Control plants. These statistical methods were used to characterise the protein spots specifically induced and repressed during S limitation, which were subsequently analysed by mass spectrometry (ESI LC–MS/MS).

## Abbreviations

2–DE: Two–dimensional electrophoresis;ACC: 1-aminocyclopropane-1-carboxylate;ANOVA: Analysis of variance;ATP: Adenosine triphosphate;C: Carbon;CHAPS: 3-[(3-Cholamidopropyl)dimethylammonio]-1- propanesulfonate;DM: Dry matter;ESI LC-MS/MS: Electrospray ionization liquid chromatography-mass spectrometry/mass spectrometry;EST: Expressed sequence tag;FNR: Ferredoxin–NADP reductase;FW: Fresh weight;GSH: Glutathione;IPG: Immobilized pH gradient;IRMS: Isotope Ratio Mass Spectrometer;LHC: Light Harvesting Complex;MDH: Malate dehydrogenase;OAS: O-acetylserine;PC: Plastocyanin;PVPP: Poly(vinylpolypyrrolidone);ROS: Reactive oxygen species;S: Sulphur;SDS: Sodium dodecyl sulphate;SE: Standard error;SOD: Superoxide dismutase;TCA: Trichloroacetic acid;WSCP: Water Soluble Chlorophyll binding Protein.

## Competing interests

The authors have declared no conflict of interest.

## Authors’ contribution

All authors contributed to the experimental design, to the plant growth and tissue sampling and have been involved in revising critically the article for important intellectual content. SE carried out S and ^34^S analyses. SE and PD performed measurements of photosynthetic activities, chlorophylls, anthocyanins and flavonols contents. PD made the other measurements and analyses, including statistical analyses and drafting the article. All authors read and approved the final manuscript.
